# Editorial: Epistemological and Ethical Aspects of Research in the Social Sciences

**DOI:** 10.3389/fpsyg.2020.00428

**Published:** 2020-03-11

**Authors:** Ulrich Dettweiler, Barbara Hanfstingl, Hannes Schröter

**Affiliations:** ^1^Department of Cultural Studies and Languages, Faculty of Arts and Education, University of Stavanger, Stavanger, Norway; ^2^Faculty of Interdisciplinary Studies, Institute of Instructional and School Development, Alpen-Adria-Universität Klagenfurt, Klagenfurt, Austria; ^3^Department “Teaching, Learning, Counselling”, German Institute for Adult Education – Leibniz Centre for Lifelong Learning, Bonn, Germany

**Keywords:** epistemology, methodology, data science, implementation science, Bayesian approach, replicability

This Research Topic focuses on the questions “behind” empirical research in the social sciences, especially in psychology, sociology and education, and presents various ideas about the nature of empirical knowledge and the values knowledge is or should be based on.

The questions raised in the contributions are central for empirical research, especially with respect to disciplinary and epistemological diversity among researchers. This diversity is also mirrored by the variety of article types collected in this issue, “Hypotheses & Theory,” “Methods,” “Conceptual Analyses,” “Review,” “Opinion,” “Commentary,” and “Book Review.”

Krueger and Heck explore in their “Hypotheses & Theory” article “The Heuristic Value of *p* in Inductive Statistical Inference.” Taking up a very lively debate on the significance of null-hypothesis testing, they explore how well the *p*-value predicts what researchers presumably seek: the probability of the hypothesis being true given the evidence, and the probability of reproducing significant results. They furthermore investigate the effect of sample size on inferential accuracy, bias, and error. In a series of simulation experiments, they find that the *p*-value performs quite well as a heuristic cue in inductive inference, although there are identifiable limits to its usefulness. Krueger and Heck conclude that despite its general usefulness, the *p*-value cannot bear the full burden of inductive inference; it is but one of several heuristic cues available to the data analyst. Depending on the inferential challenge at hand, investigators may supplement their reports with effect size estimates, Bayes factors, or other suitable statistics, to communicate what they think the data say.

The argumentation of this article is flanked with a “Comment” on the article “The Need for Bayesian Hypothesis Testing in Psychological Science” (Wagenmakers et al., 2017) by Perezgonzalez. He argues that Wagenmakers et al. fail to demonstrate the illogical nature of *p*-values, while, secondarily, they succeed to defend the philosophical consistency of the Bayesian alternative. He comments on their interpretation of the logic underlying *p*-values without necessarily invalidating their Bayesian arguments. A second contribution by Perezgonzalez et al. deals with a comment on epistemological, ethical, and didactical ideas to the debate on null hypothesis significance testing, chief among them ideas about falsificationism, statistical power, dubious statistical practices, and publication bias presented by Heene and Ferguson (2017). The authors of this commentary conclude that frequentist approaches only deal with the probability of data under H0 [*p*(*D*|*H*_0_)]. If anything about the (posterior) probability of the hypotheses is at question, then a Bayesian approach is needed in order to confirm which hypothesis is most likely given both the likelihood of the data and the prior probabilities of the hypotheses themselves.

Hanfstingl argues in her “Hypotheses & Theory” article “Should We Say Goodbye to Latent Constructs to Overcome Replication Crisis or Should We Take Into Account Epistemological Considerations?”, that a lack of theoretical thinking and an inaccurate operationalization of latent constructs leads to problems that Martin Hagger calls “déjà variables,” which ultimately also contribute to a lack of replication power in the social sciences. She proposes to use assimilation and accommodation processes instead of induction and deduction to explicate the development and validation of latent constructs and theories.

In the “Methods” article “On the development of a computer-based tool for formative student assessment: epistemological, methodological and practical questions,” Tomasik et al. present a computer-based tool for formative student assessment. They deal with epistemological and methodological challenges as well as challenges in the practical implementation of these instruments. Overall, the authors show how formative assessment can not only increase efficiency, but also increase the validity of such feedback processes.

Closely related to this topic is the “Review” article by Moir. She defines components necessary to promote authentic adoption of evidence-based interventions and assessments in education, thereby increasing their effectiveness and investigates, how the quality of implementation has directly affected the sustainability of two such successful interventions. By analyzing implementation science, some of the challenges currently faced within this field are highlighted and areas for further research discussed. Furthermore, this article links to the implications for educational psychologists and concludes that implementation science is crucial already to the design and evaluation of interventions, and that the educational psychologist is in an ideal position to support sustainable positive change.

In “Linearity vs. Circularity? On Some Common Misconceptions on the Differences in the Research Process in Qualitative and Quantitative Research,” Baur discusses the exaggeratedly simplified distinction between quantitative and qualitative paradigms in research methods and explains why we must assume a fluent transition between the two approaches. She points to similarities between the two supposedly antagonistic approaches in the use of induction, deduction and abduction, the roundness of the applied research phases and the analyses performed.

Closely related to that article, Dettweiler argues in his “Opinion” article that in both, so-called qualitative and quantitative research, it is inevitable for the research to define his or her prior beliefs, and that it is deeply irrational to believe that research methods are purely formal, distinct and free from value-judgements. There is also an informal part inherent to rationality in science which depends on the changing beliefs of scientists (Dettweiler).

Another “Opinion” article deals with some ethical challenges with pre-registration. Yamada argues that pre-registration, which should secure the transparency in the research process, including the experimental and analytical methods, the researchers' motivation and hypotheses, can easily be “cracked.” She introduces the idea that to prevent such cracking, registered research reports should not be completely accepted as secure and valid just because “they were registered”; instead, several replications of the reported research with pre-registration should be performed. In addition, outsourcing experiments to multiple laboratories and agencies that do not share profitable interests with those of the registered researchers can be an effective means of preventing questionable research practices.

Where, Yamada refers to replication as a remedy to questionable research practice, Bressan presents a “Conceptual Analysis” and puts her finger into such questionable practice in the “Open Science Collaboration's Reproducibility Project,” where a replication proved to be confounded. She shows in a case study on a “failed replication” that the dataset contained a bias which was absent in the original dataset; controlling for it replicated the original study's main finding. She concludes that, before being used to make a scientific point, all data should undergo a minimal quality control. Because unexpected confounds and biases can be laid bare only after the fact, we must get over our understandable reluctance to engage in anything *post-hoc*. The reproach attached to *p*-hacking cannot exempt us from the obligation to (openly) take a good look at our data.

In her contribution “Quantitative Data From Rating Scales: Quantitative Data From Rating Scales: An Epistemological and Methodological Enquiry,” classified as a “Methods” type article, Uher presents yet another perspective on the “replication crisis” and fundamentally criticizes some traditions of psychological measurement and evaluation. Referring to the Transdisciplinary Philosophy-of-Science Paradigm for Research on Individuals (TPS Paradigm), she investigates psychological and social science concepts of measurement and quantification. Uher proposes to apply metrological measurement concepts with a more precise focus on data generation.

Lastly, a “Book-review” by Perezgonzalez et al. on “Another science is possible: a manifesto for slow science” (Stengers and Muecke, 2018) is completing this collection.

We sincerely hope that this collection can in fact contribute to such “another science,” a science that does not build on shallow dichotomies, such as “qualitative” or “quantitative,” a science that is transparent, rigorous, epistemologically informed, and ethical.

## Author Contributions

All authors listed have made a substantial, direct and intellectual contribution to the work, and approved it for publication.

### Conflict of Interest

The authors declare that the research was conducted in the absence of any commercial or financial relationships that could be construed as a potential conflict of interest.

## References

[B1] Heene M. Ferguson C. J. (2017). Psychological science's aversion to the null, and why many of the things you think are true, aren't, in Psychological Science Under Scrutiny: Recent Challenges and Proposed Solutions, eds Lilienfeld S. O. Waldman I. D. (Chichester: John Wiley & Sons), 34–52.

[B2] Stengers I. Muecke S. (2018). Another Science Is Possible : A Manifesto for Slow Science (English edition. ed.). Cambridge: Polity.

[B3] Wagenmakers E. J. Verhagen J. Ly A. Matzke D. Steingroever H. Rouder J. N. (2017). The need for Bayesian hypothesis testing in psychological science, in Psychological Science Under Scrutiny: Recent Challenges and Proposed Solutions (Hoboken, NJ: Wiley-Blackwell), 123–138.

